# Hyperbaric oxygen therapy for wound healing in diabetic rats: Varying efficacy after a clinically-based protocol

**DOI:** 10.1371/journal.pone.0177766

**Published:** 2017-05-17

**Authors:** Johan W. van Neck, Bastiaan Tuk, Esther M. G. Fijneman, Jonathan J. Redeker, Edwin M. Talahatu, Miao Tong

**Affiliations:** Department of Plastic and Reconstructive Surgery, Erasmus MC—University Medical Center, Rotterdam, the Netherlands; NYU Langone Medical Center, UNITED STATES

## Abstract

Hyperbaric oxygen therapy (HBOT) is a clinical treatment in which a patient breathes pure oxygen for a limited period of time at an increased pressure. Although this therapy has been used for decades to assist wound healing, its efficacy for many conditions is unproven and its mechanism of action is not yet fully clarified. This study investigated the effects of HBOT on wound healing using a diabetes-impaired pressure ulcer rat model. Seven weeks after streptozotocin-induced diabetes in rats (n = 55), a pressure ulcer was created on dorsal skin. Subsequently, animals received HBOT during 6 weeks following a standard clinical protocol (HBOT group with varying endpoints up to 42 days post-wounding) versus controls without HBOT. Capillary venous oxygen saturation (SO_2_) showed a significant increase in the HBOT group on day 24; however, this increase was significant at this time point only. The quantity of hemoglobin in the micro-blood vessels (rHB) showed a significant decrease in the HBOT group on days 21 and 42, and showed a trend to decrease on day 31. Blood flow in the microcirculation showed a significant increase on days 17, 21 and 31 but a significant decrease on days 24 and 28. Inflammation scoring showed significantly decreased CD68 counts in the HBOT group on day 42, but not in the early stages of wound healing. Animals in the HBOT group showed a trend for an increase in mean wound breaking strength on day 42.

## Introduction

The treatment of impaired wound healing remains a challenge. Various biological processes, such as chronic inflammation, hampered keratinocyte functioning, and abnormalities in growth factor production, extracellular matrix deposition and in the tissue remodeling process, are proposed to contribute to compromised diabetes-impaired wound healing [1–4].

Hyperbaric oxygen therapy (HBOT) is a clinical treatment modality in which a patient breathes pure oxygen while exposed to an increased atmospheric pressure of (generally) 2.4 atmospheres absolute (ATA) [5, 6]. HBOT is used to treat refractory diabetic wounds and reduce the risk of lower extremity amputation, with the aim to improve quality of life and reduce the overall costs of care [7, 8].

The main mechanism of HBOT is thought to be related to the oxidative stress response that improves neovascularization [6]. After HBOT, cells in the wound area exhibit increased growth factor production and neovascularization, as well as improved cell migration and collagen synthesis. A separate, free radical-based mechanism for the augmentation of neovascularization by HBOT is through circulating stem/progenitor cells (SPCs). Hyperoxia stimulates mobilization of bone marrow SPCs and improves their function once they home to peripheral sites [9].

Although HBOT is recommended in the treatment of diabetic wounds, its clinical effectiveness remains unclear for many conditions. Moreover, in diabetic foot ulcers, HBOT increased the rate of early ulcer healing but failed to provide a benefit in wound healing on long-term follow-up [8]. Also, there is no evidence that HBOT promotes healing of venous, arterial and pressure ulcers [8].

Studies on the efficacy and mechanisms of action of HBOT in a clinical setting have certain limitations, whereas experimental animal models allow in-depth laboratory analysis. In the present study, a standard clinical protocol was used: the animals receiving HBOT were exposed to 100% oxygen for 1.5 h at 2.4 ATA, during weekdays for 6 weeks [6].

Our earlier study on the effects of 4 weeks HBOT in rats that were diabetic for one month, showed improved oxygen saturation of the lower end of the capillary network together with increased mean skin breaking strength; however, compared with controls, the difference did not reach statistical significance [10].

The present study tests our hypothesis that HBOT delivers increased amounts of oxygen to the wound area, leading to enhanced wound healing and improved tissue restoration. The efficacy of HBOT was evaluated in pressure ulcers generated in rats that had untreated streptozotocin (STZ)-induced diabetes for 7 weeks before wounding and HBOT. Effects of HBOT were investigated up to 42 days post-wounding and consisted of evaluating the wound area, tissue oxygen supply, vasodilatory capability, inflammation, neovascularization and tissue breaking strength.

## Materials and methods

### Animals

WAG/RijHsd female rats (n = 55, 8 weeks old), SPF, were purchased from Charles-River (L’Arbresle, France). The rats were housed with two animals per cage, exposed to a 12-h light/dark cycle (lights on period: 7 AM-7 PM), at a temperature of 21–23°C and fed with a standard laboratory diet (Hope Farms, Woerden, the Netherlands) with food and water available ad libitum. Cage enrichment was applied. During the experimental procedures, rats were inhalation-anesthetized using 1.5% isoflurane (Pharmachemie BV, Haarlem, the Netherlands) in oxygen as carrier.

The experimental protocol was approved by an Animal Experiments Committee (Stichting Dec-Consult, the Netherlands), under the National Experiments on Animals Act and adhered to the rules laid down in this national law that serves the implementation of Guidelines on the Protection of Experimental Animals by the Council of Europe (1986), Directive 86/609/EC.

### Induction of diabetes

After overnight fasting, rats were injected with STZ (Sigma-Aldrich, St. Louis, MO, USA), intraperitoneal, at a dose of 60 mg/kg body weight in 0.05 mol/L sodium citrate buffer, pH 4.5. Blood glucose was determined in the fed state at 9 AM (Zeitgeber time ZT2) using a OneTouch glucometer (LifeScan, Milpitas, CA, USA), twice weekly during the first 3 weeks, followed by a weekly measurement. All animals had blood glucose levels ≥ 20 mmol/L throughout the experimental period.

### Ulceration model and HBOT

A total of 55 diabetic rats were ulcerated, on average 7 weeks after STZ injection. The ulcer is created by clamping two-magnet-disk (15 mm diameter each) on the dorsal skin of the rat. The clamping duration (ischemic period) is 16 h, which created two 15-mm diameter ulceration wounds. During the ischemic period and for three days post-clamping the rats received analgesia (Temgesic 0.5 mg/kg; Reckitt Benkiser Pharmaceuticals, Berkshire, UK).

After the ischemic period animals were randomly divided into three groups: Group 1, endpoint day 7 (n = 18); Group 2, endpoint day 14 (n = 18); and Group 3, endpoint day 42 (n = 19). Within each group the animals were divided into the HBO-treated group and the control group, all having comparable glucose levels and body weight. HBO-treated rats were given 100% oxygen under a pressure of 2.4 ATA for 90 min, using a custom designed hyperbaric oxygen tank (IHC High Tech, Raamsdonkveer, the Netherlands) suitable for animals [11]. Non-HBOT (control) rats experienced similar handling and similar machine noise as the HBOT animals, but breathed normoxia at sea level pressure.

### Macroscopic analysis

Body weight measurement and ulcer photography were performed weekly. A ruler was placed next to the wound as calibration for further analyses. The macroscopic wound areas were calculated using ImageJ (National Institutes of Health, Bethesda, Maryland, USA).

### Sampling and preparation

When reaching their respective experimental endpoints, the rats were anesthetized and euthanized by thoracic bleeding. For rats assigned for breaking strength measurements, the dorsal pelt was excised. For all other rats, the ulcer tissues, including 2 mm of the surrounding normal skin, was excised and cut into halves. One half was fixed in 10% phosphate-buffered formalin for histology and immunohistochemistry. From the other half of the wound, the surrounding normal skin tissue was excised and the wounded tissue was snap-frozen in liquid nitrogen and homogenized using a Mikro-Dismembrator (B. Braun Biotech International, Melsungen, Germany).

### Breaking strength measurements

Breaking strength was measured as described previously [12]. In brief, the skin containing the wound area was excised and cut into an hour glass-shaped strip with standard dimensions of 4 x 45 mm. The strips were lengthwise positioned in a 10-kg force transducer that is part of a Testometric® AX, M250-2.5KN tensiometer (Testometric Co. Ltd, Lancashire, UK). Breaking strength is measured by determining the failure force of the skin strip.

### Immunohistochemistry

To evaluate inflammation and angiogenesis in the ulcer tissue, CD68 (a monocyte/macrophage marker, purchased from AbD Serotec, Düsseldorf, Germany), CD34 (an endothelial cell marker, purchased from R&D Systems, Minneapolis, MN, USA) and a vascular endothelial growth factor (VEGF; purchased from AbD Serotec, Düsseldorf, Germany) were used, respectively, and detected using immunohistochemistry (ABC complex), as described previously [13]. Stained and mounted sections of HE, CD34 and CD68 were scanned for virtual microscopy at 40x magnification, using a Hamamatsu Nanozoomer 2.0-HT robot (Hamamatsu Photonics K.K., Hamamatsu City, Japan). Subsequently, using virtual microscopy software NDP (Hamamatsu), JPEG images of all sections except HE, were taken at 2.5X magnification (10x digital zoom) and loaded into CellD software (Olympus, Hamburg, Germany) for computerized quantitative analysis. Thresholds for number of pixels, hue, saturation, and intensity were set and verified by human eye. Either percentage staining of the total area (CD34, CD68) or the absolute dermal thickness was calculated.

### Vasodilatory capability measurements

Laser Doppler Flowmetry (LDF) with local heat provocation measures the vasodilatory capabilities of the wounded skin following super physiological local heating to 44°C. The microcirculatory response to this local heating was performed at day 42, the end point of the study, and monitored as described in detail previously [12].

### Tissue oxygen supply

Oxygen supply in the microcirculation was measured using an O2C Laser Doppler Flow meter and tissue spectrometer (LEA Medizintechnik Giessen, Germany). Oxygen saturation of hemoglobin (SO_2_), was measured at the venous end of the capillaries; this point represents the lowest oxygen saturation of the tissue. The relative amount of hemoglobin (rHB) represents the quantity of hemoglobin in the micro-blood vessels and, therefore, reflects the density of the blood vessels [14].

### Statistical analysis

Analyses were performed with IBM SPSS software, version 21 (Chicago, IL, USA). An independent samples t-test was used to compare results between the two groups. Two-sided p-values ≤ 0.05 were considered to indicate statistically significant differences. Treatment differences over time were analyzed using a model of general linear repeated measures. Data are presented as means ± SEM.

## Results

### Diabetes induction and glucose levels

Female WAG/RijHsd rats (n = 55), on average 8 weeks old and with an average weight of 134.4 ± 7.9 g, were used in the study. Following STZ injection, all rats became hyperglycemic: 50 rats had blood glucose levels > 19 mmol/L in the period after the STZ injection until their experimental endpoint. In five rats the blood glucose levels gradually dropped towards the end of the experiment; however, these animals still had blood glucose levels > 10 mmol/L at all times during their experimental period. In four rats, blood glucose levels dropped to normoglycemia in the diabetes induction period; these animals were reinjected with STZ and then maintained glucose levels of >20 mM during the remainder of the study protocol.

All 55 rats showed similar behavior and were included in the analysis. No significant differences in mean blood glucose level were observed between the two groups during the treatment period.

### Effect of diabetes and HBOT on body weight

The average loss of body weight during the first week after STZ injection was 4% (average weight 130 g). At the end of the diabetes induction phase (week 7) the average body weight had increased to 104% of the starting weight (average weight 140 g) with no significant differences between the groups assigned to the subsequent HBOT or control treatment.

Analysis of the body weight data during the experimental phase on a per-group basis showed no significant differences in body weight between the HBOT and control groups that had their experimental endpoint on day 7 and day 14.

On an average of 7 weeks after diabetes induction, two pressure ulcer wounds were created on the dorsal skin of each animal. During the wound-healing phase, HBOT rats showed a significantly increased body weight in experimental week 4 (HBOT 146 ± 3 vs. control 136 ± 2 g; 95% confidence interval [CI] 3–18 g; p = 0.009) and in experimental week 5 (HBOT 149 ± 3 vs. control 141±2 g; 95% CI 1–14 g; p = 0.026).

### Effects of HBOT on wound area reduction

**Fig 1:**
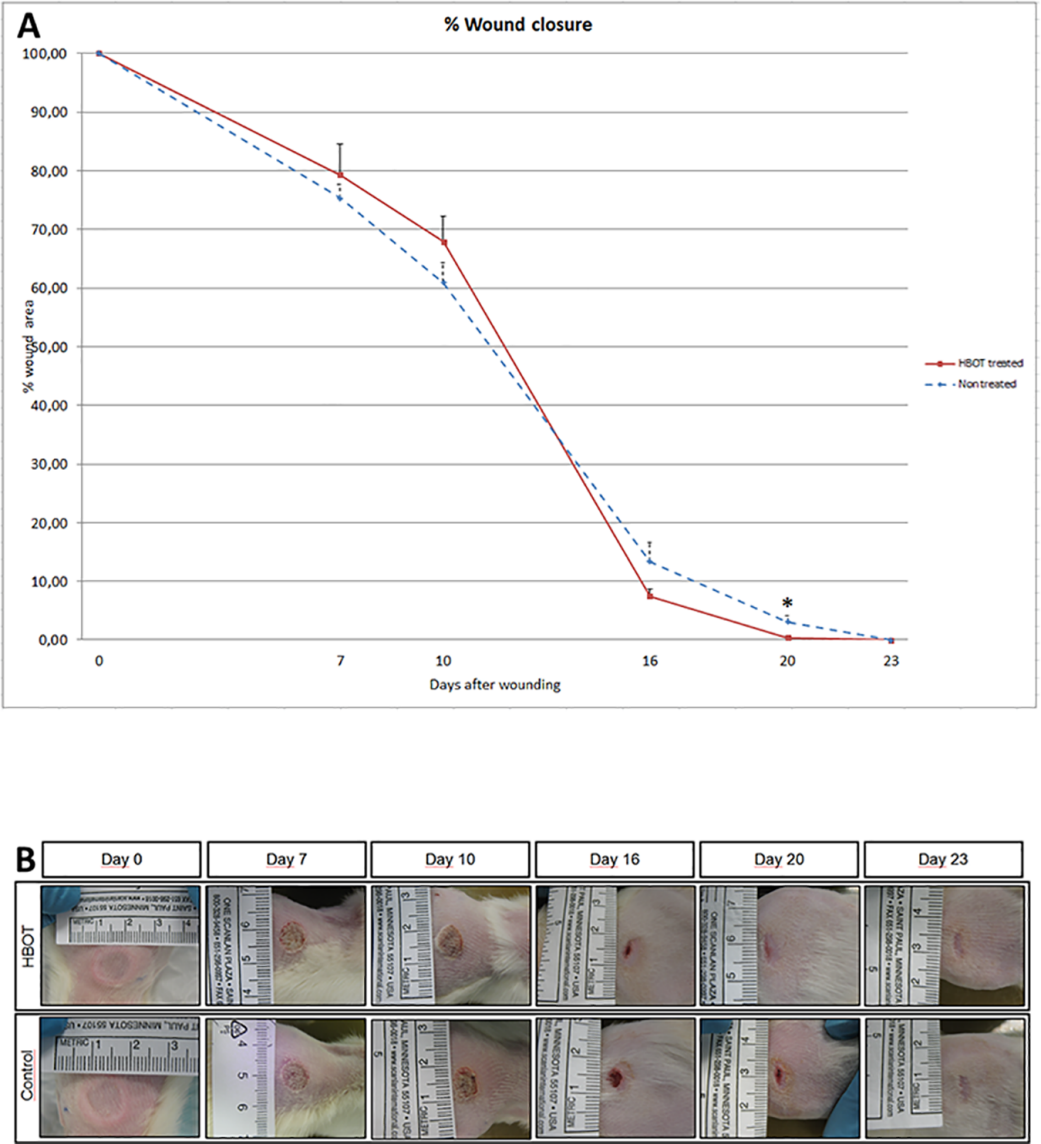
Effect of HBOT on wound area reduction. **A.** The effect of HBOT on wound area reduction was measured using ImageJ. The measured wound area at day 0 was set to 100%. Data are presented as mean ± SEM. *p < 0.05 indicates a significant difference between the HBOT and control group. **B.** Representative macroscopic images of wounds from HBOT and control animals on experimental days 0, 7, 10, 16, 20 and 23.

Fig 1A shows that the reduction in the mean wound area was delayed in the HBOT group on day 10 post-wounding compared to the control group; however, this difference was not significant (67.9 ± 4.4% versus 60.9 ± 3.4%; p < 0.23). On experimental day 16, the mean wound area in the HBOT group was reduced to 7.4 ± 1.3% vs. 13.4 ± 3.3% in the control group (p < 0.09) and reached a significant reduction on experimental day 20 (HBOT 0.4 ± 0.2% vs. control 3.1 ± 1.1%; 95% CI 0.3–5.1%; p = 0.028). However, analysis of the treatment effects over time (day 0 to day 23) showed no significant difference in wound area reduction between the two groups. Representative images of the wounds are presented in Fig 1B.

### Tissue oxygen supply

Tissue oxygen levels were determined in the HBOT and control groups at multiple time points and started on day 17 post-wounding, i.e. the moment when the eschar had detached from all wounds.

**Fig 2:**
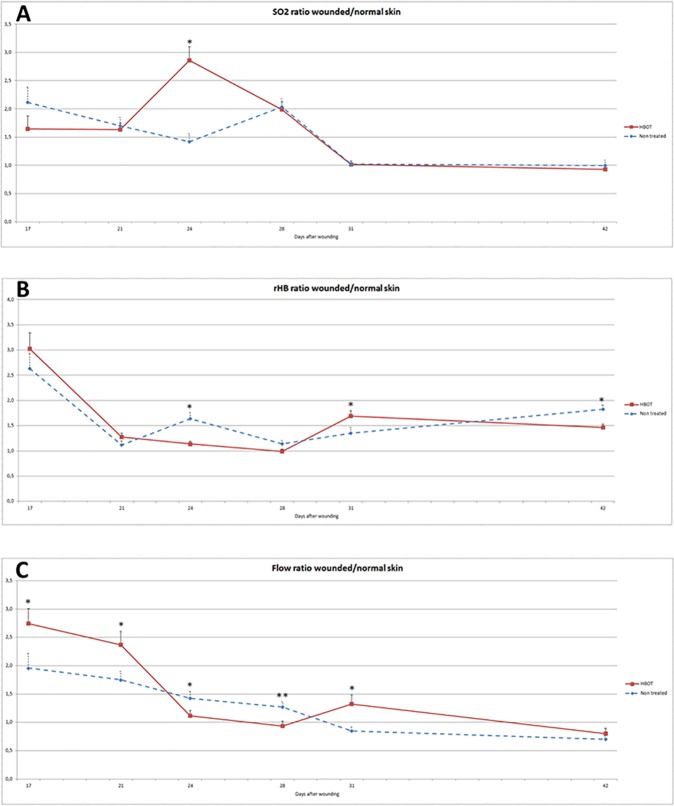
Effect of HBOT on wound tissue perfusion. **A. Effect of HBOT on hemoglobin oxygen saturation at the venous end of the capillaries (SO**_**2**_**)** Oxygen saturation in the wounded and normal skin was measured in the HBOT and control animals (20 h post-HBOT). Oxygen saturation in the wound is expressed as a percentage of the flow in the normal skin of the same animal. **B. Effect of HBOT on quantity of hemoglobin.** Quantity of hemoglobin in the wounded and normal skin was measured in the HBOT and control animals (20 h post-HBOT). Quantity of hemoglobin in the wound is expressed as a percentage of the flow in the normal skin of the same animal. **C. Effect of HBOT on blood flow.** Blood flow in the wounded and normal skin was measured in the HBOT and control animals (20 h post-HBOT). Blood flow in the wound is expressed as a percentage of flow in the normal skin of the same animal. Data are shown as means ± SEM. *p ≤ 0.05, **p ≤ 0.01 and ***p ≤ 0.001 indicate significant differences between the HBOT and control group.

The capillary-venous oxygen saturation (SO_2_) (Fig 2A), the relative amount of hemoglobin (rHB) (Fig 2B), and blood flow in the microcirculation (Fig 2C) were determined. To correct data for possible between-animal variation in tissue architecture and vascular characteristics, oxygen supply was measured in the wounds and normal skin of the same animal. Fig 2 presents the values in the wound as a percentage of skin perfusion in non-wounded skin.

Capillary-venous oxygen saturation showed no difference between the two groups except on day 24, when the SO_2_ ratio was increased two-fold in HBOT animals as compared to control animals (2.86 ± 0.24 in the HBOT group vs. 1.42 ± 0.14 in the control group; 95% CI 0.87–2.01; p = 0.000) (Fig 2A). No explanation could be found for this significant difference at this single time point. Analysis of the treatment effects over time (day 7 to day 42) showed no significant difference in tissue oxygen supply between the two groups.

The quantity of hemoglobin in the micro-blood vessels (rHB) showed a significant decrease in the HBOT group on day 24 (1.14 ± 0.05 in the HBOT group vs.1.64 ± 0.12 in the control group; 95% CI 0.25–0.75; p = 0.000) and on day 42 (1.46 ± 0.07 in the HBOT group vs. 1.83 ± 0.08 in the control group; 95% CI 0.16–0.57; p = 0.001) but showed a significant increase on day 31 in this group (1.69 ± 0.10 in the HBOT group vs. 1.35 ± 0.1 in the control group; 95% CI 0.67–0.004; p = 0,048) (Fig 2B).

Flow in the microcirculation showed a significant increase in the HBOT group on day 17 (2.74 ± 0.26 in the HBOT group vs. 1.96 ± 0.26 in the control group; 95% CI 1.53–0.04; p = 0.04) and day 21 (2.37 ± 0.23 in the HBOT group vs. 1.75 ± 0.15 in the control group; 95% CI 1.18–0.05; p = 0.03), a significant decrease on day 24 (1.12 ± 0.09 in the HBOT group vs. 1.43 ± 0.12 in the control group; 95% CI 0.62–0.00; p = 0.05) and day 28 (0.94 ± 0.08 in the HBOT group vs. 1.27 ± 0.10 in the control group; 95% CI 0.59–0.07, p = 0.01), and increased again on day 31 (1.33 ± 0.16 in the HBOT group vs. 0.85 ± 0.07 in the control group; 95% CI 0.83–0.13, p = 0.03) (Fig 2C).

### Effects of HBOT on granulation tissue deposition

**Fig 3:**
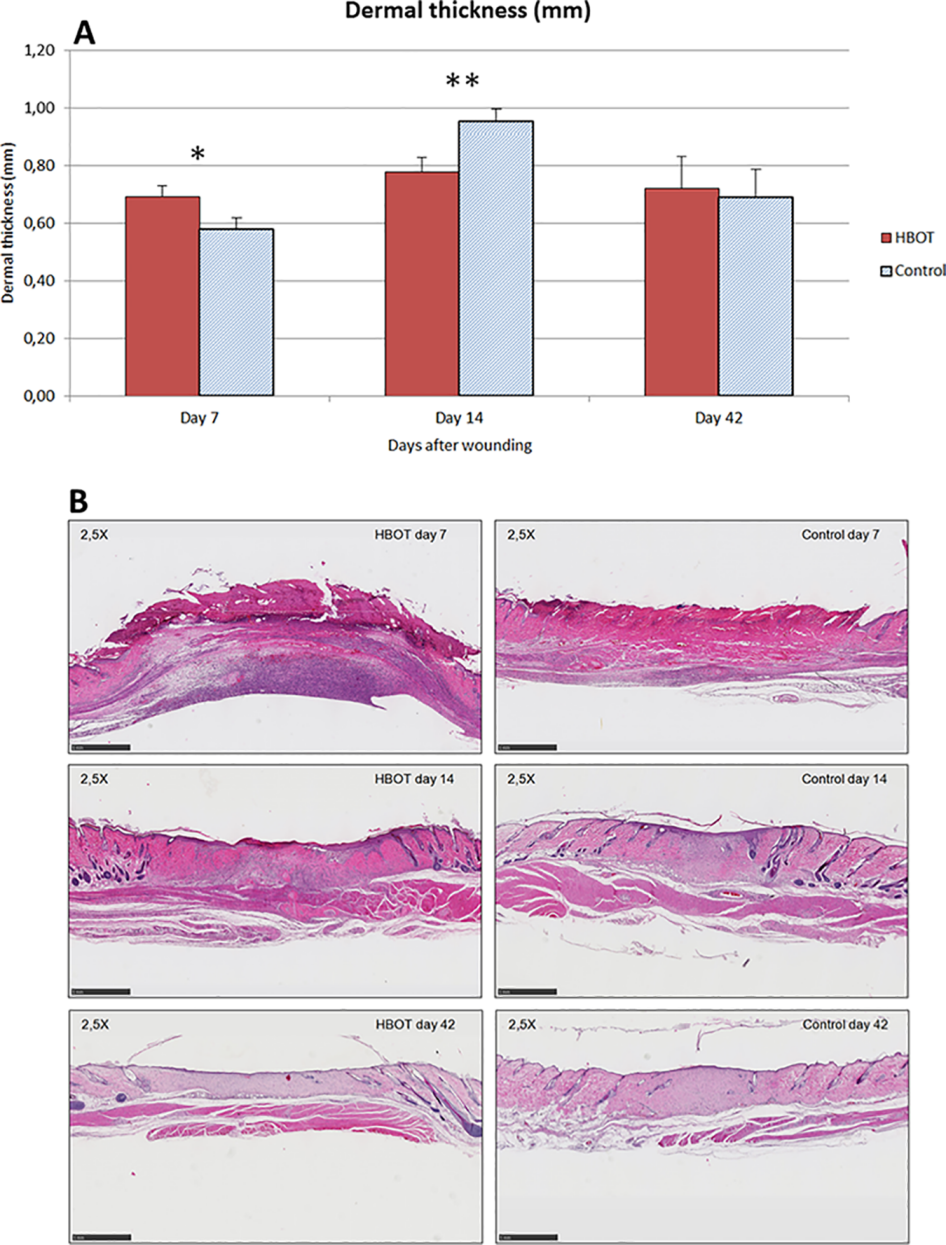
**A. Effect of HBOT on formation of granulation tissue.** The effect of HBOT on the formation of granulation tissue (or neo-dermis) was assessed by H&E stained histology and CellD measurement at the experimental endpoints. Data are presented as mean ± SEM. *p ≤ 0.05, and **p ≤ 0.01 indicate significant differences between the HBOT and control group. **B**. Representative images of the wound histology of HBOT and control groups on experimental days 7, 14 and 42. Scale bar: 500 μm.

The formation of granulation tissue (or neo-dermis) was measured on hematoxylin and eosin (H&E) stained sections on all experimental end points. HBOT caused a significant increase in granulation tissue deposition in the wound area on day 7 (0.69 ± 0.04 mm in the HBOT group vs. 0.58 ± 0.04 mm in controls, 95% CI -0.22 to 0.00 mm; p = 0.04). However, on day 14, the neo-dermal thickness of the wounds in the control group showed a significant increase (0.78 ± 0.05 in the HBOT group vs. 0.95 ± 0.04 mm in the control group, 95% CI 0.04–0.31 mm; p = 0.01), whereas on day 42 no significant differences were observed between the two groups (Fig 3A). Epidermal measurements in the wound area showed no significant difference between the HBOT and control group. Representative images of the wound histology are presented in Fig 3B.

### Effects of HBOT on inflammation

**Fig 4:**
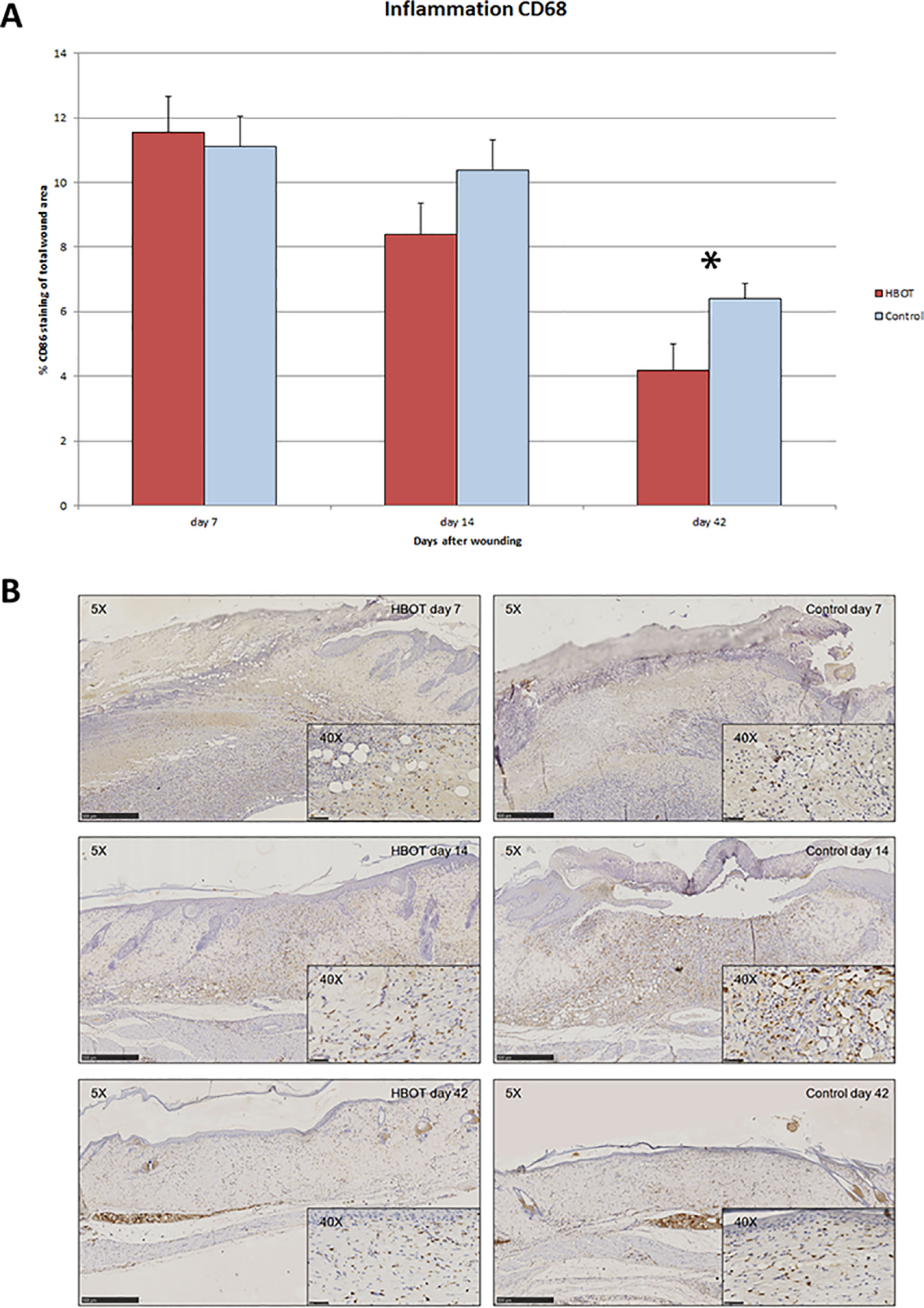
**A. Effect of HBOT on inflammation reduction.** The effect of HBOT on the inflammatory cell influx was evaluated by quantitating the immunohistochemical stain of the monocyte/macrophage marker CD68. Data are presented as mean ± SEM. *p ≤0.05 indicates a significant difference in inflammatory response between the HBOT and control group.**B.** Representative images of CD68 wound immunohistochemistry of HBOT and control animals on experimental days 7, 14 and 42. Scale bar: 500 μm. Scale bar 40x insert: 50 μm.

Inflammation was evaluated by determining the abundance of monocytes/macrophages in the wounds using an anti-CD68 antibody. Representative immunohistochemical images are presented in Fig 4B. As shown in Fig 4A, on day 42 the score of CD68 staining was significantly reduced in the HBOT wounds (4.18 ± 0.83 in the HBOT group vs. 6.39 ± 0.49 in the controls, 95% CI 0.96–4.33; p = 0.04). However, analysis of the treatment effects over time (day 7 to day 42) showed no significant difference in the abundance of monocytes/macrophages in the wounds between the two groups.

### Effects of HBOT on vascularization

**Fig 5:**
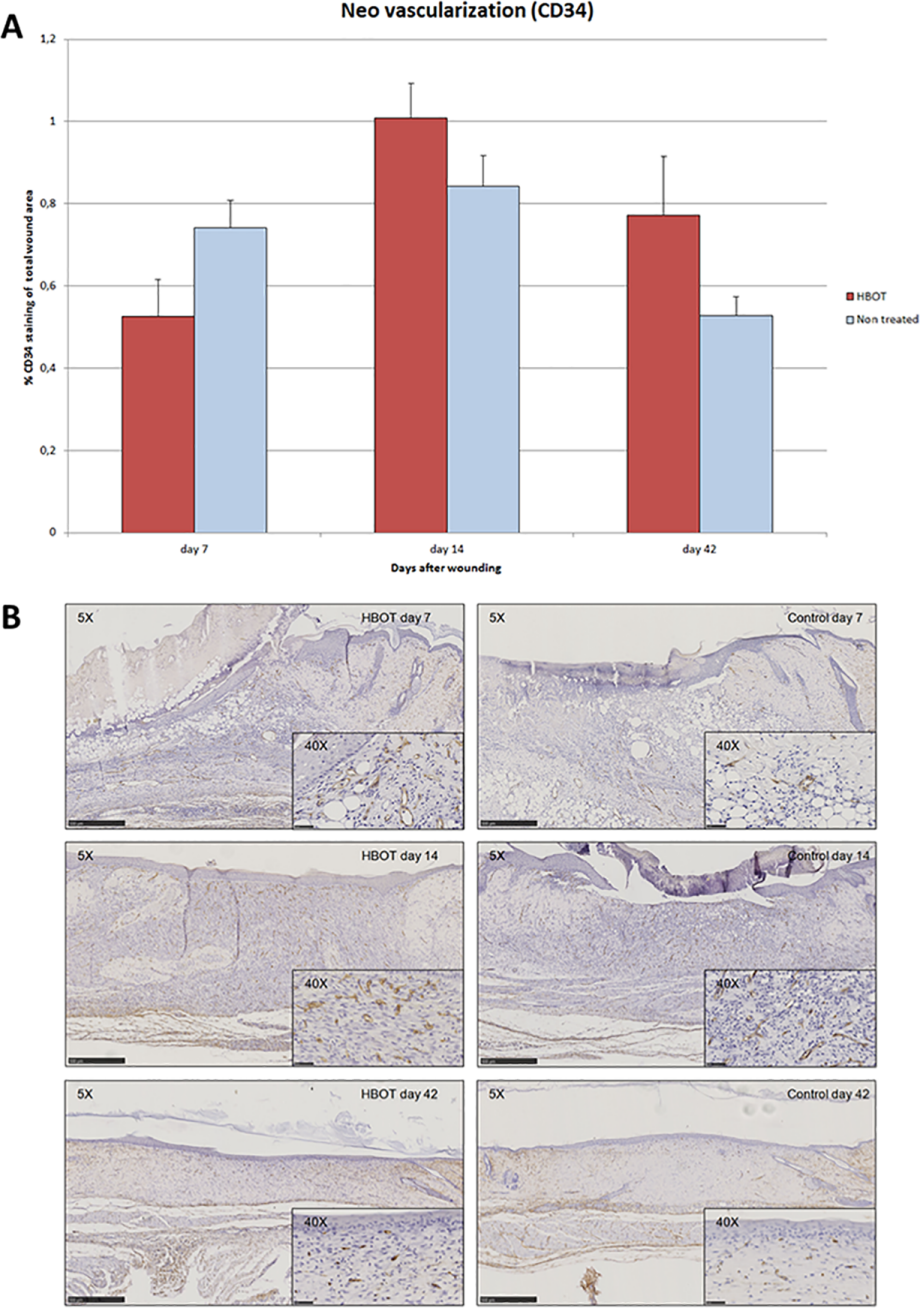
**A. No significant effect of HBOT on enhancing vascularization.** The effect of HBOT on the process of vascularization was evaluated by the immunohistochemical stain of the endothelial cell marker CD34. Data are presented as mean ± SEM. **B**. Representative images of CD34 wound immunohistochemistry of HBOT and control animals on experimental days 7, 14 and 42. Scale bar: 500 μm. Scale bar 40x insert: 50 μm.

Vascularization was visualized by immunostaining the endothelial cells in the wound tissue using the endothelial cell marker CD34. The morphology of CD34 positive endothelial cells could easily be discriminated from residual hematopoietic stem cells. No significantly different vascularization could be observed between the HBOT and control group although a trend towards increased vascularization by HBOT could be observed at days 14 (HBOT 1.01±0.08 vs. controls 0.84±0.08, 95% CI -0.39 to 0.06; p = 0.14) and 42 (HBOT 0.77±0.14 vs. controls 0.53±0.05, 95% CI -0.57 to 0.08; p = 0.12) (Fig 5A). Representative immunohistochemical images are presented in Fig 5B.

### Effects of HBOT on vasodilatory response to local heating

**Fig 6:**
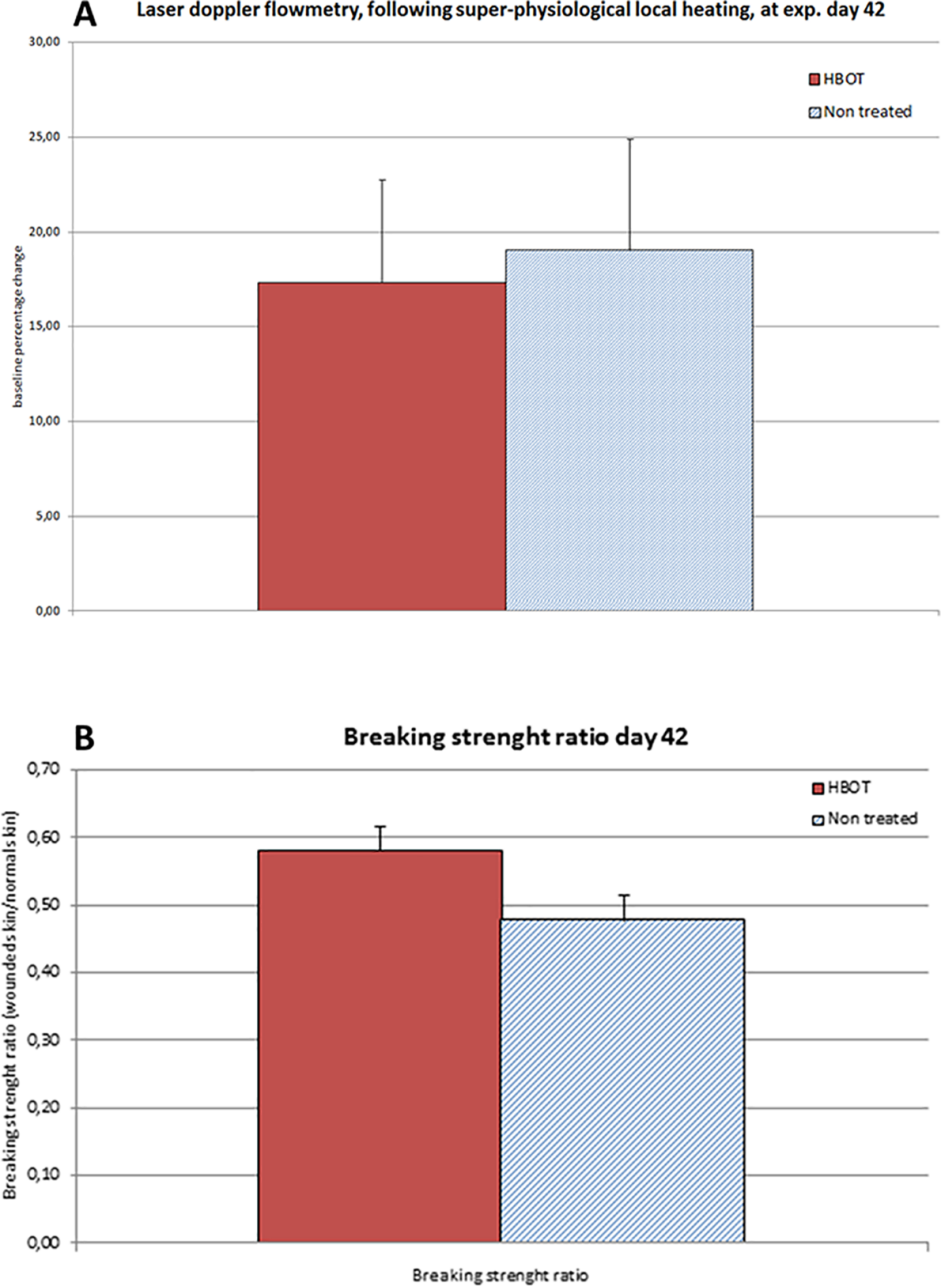
**A. Effect of HBOT on the vasodilatory response to super-physiological local heating on day 42 post-wounding.** The vasodilatory response to local heating (44°C for 10 min) was measured in the wounds of HBOT and control animals by laser doppler flowmetry. The vasodilatory response in the wound tissue is expressed as the increase in blood flow over baseline flow. **B. Ratio of the skin breaking strength of normal and wounded skin in diabetic rats on day 42 post-wounding.** Breaking strength of the wounded skin is depicted as a percentage of the breaking strength of the normal skin in the same animal. The wounded skin versus normal skin ratio was calculated for the HBOT and control group on day 42. Data are presented as mean ± SEM.

At the experimental endpoint at day 42, the wounds were monitored for their vasodilatory response to super-physiological local heating using LDF. No differences in excess heat provoked perfusion characteristics were observed between the HBOT and control groups (percentage baseline change HBOT 19.1 ± 5.8% vs. control 17.3 ± 5.4%; p = 0.84) (Fig 6A).

### Effects of HBOT on tissue breaking strength

At the experimental endpoint on day 42, HBOT showed no significant effect on the breaking strength of wounded skin (20.0 ± 1.2 N in the HBOT group vs. 17.3 ± 1.2 N in the control group; NS) and unwounded skin (34.6 ± 1.4 in the HBOT group vs. 36.7 ± 1.0 in the controls; NS). However, when the breaking strength of the wounded tissue was expressed as a percentage of the strength of the surrounding unwounded skin, the HBOT showed an increased mean value (0.58 ± 0.04 of normal skin in the HBOT group vs. 0.48 ± 0.04 in the control group, 95% CI -0.21 to 0; p = 0.065) (Fig 6B).

## Discussion

The treatment of diabetic ulcers requires a multifactorial approach due to the multifactorial etiology of diabetic ulcers [15]. The approach includes an extensive debridement, off-loading of areas with high pressure, control of metabolism and concomitant diseases, ulcer care, and the education of care. However, despite applying all conventional treatment methods, the treatment of diabetic ulcers often fails to match the expectations of healthcare professionals. Then, adjunctive therapies, such as topically and systemically applied growth factors, bioengineered biological coverings, and HBOT, are considered. However, evidence for the effectiveness of most of these therapies, including HBOT, is scarce [8, 16].

As an adjunctive treatment of diabetic ulcers HBOT has been used for over 40 years, but its efficacy remains controversial. Due to the limited evidence in randomized controlled trials (RCTs), it is difficult to conclusively support or reject the benefits of HBOT in treating diabetic ulcers [17–20]. No significant effects on amputation rates have been found in RCTs evaluating HBOT (19). For diabetic foot ulcer healing, although positive results have been reported [7, 8, 21, 22], the rationale behind the observed effect is sometimes unclear. For example, in the RCT of Londahl et al. [22], a significant effect of HBOT on ulcer size reduction was observed only from experimental month 9 onwards; this is 7 months after the last HBOT session. In another study, a period of almost 1 year between cessation of HBOT and a significant effect was reported [23]. To elucidate the effects of HBOT, insight into several parameters and tissue response is needed that, for ethical reasons, cannot easily be achieved in studies among patients.

In the present study, a STZ-induced diabetic rat model of ischemia-reperfusion injury-induced pressure ulcers was used in which the healing time of magnet compression-induced ulcers was delayed by over 50% [13, 24]. The HBOT protocol used in the present study follows standard clinical practice. The outcomes of wound closure, tissue oxygen supply, vasodilatory capability, inflammation, neovascularization, and tissue breaking strength, were evaluated; however, most of the results obtained are not straightforward.

The magnet clamping ulceration model creates extensive vascular damage. All blood vessels beneath the magnet compression area become necrotic, which calls for extensive neovascularization. Achieving increased neovascularization is an important rationale for using HBOT [23, 25]. In our animal model, neovascularization in ulcer tissue was found to be increased after HBOT at the early stage of wound healing. HBOT also stimulated granulation tissue formation on day 7; however, this effect was reversed on day 14. Neovascularization is involved in both angiogenesis and vasculogenesis. Regional growth factors stimulate the former, and recruitment and differentiation of circulating SPCs stimulate the latter. In the present study, the production of growth factors such as VEGF (the most specific growth factor for neovascularization) was not enhanced (data not shown). Therefore, the mechanism of action of HBOT in increasing neovascularization is probably not directly related to angiogenesis.

In the literature, two potentially additive mechanisms of HBOT action have been proposed.

1. HBOT transports oxygen to bodily sites where vascularization is poor or absent, such as in poorly healing wounds. This proposed mechanism of HBOT action relates to the physical relationship between pressure and gas concentration in a liquid [26]. It is known that 2.4 ATA of pure oxygen dissolves a substantial amount of oxygen in blood plasma. In the late 1950s, Boerema et al. showed that phlebotomized dogs could survive in HBOT conditions [27].

2. Cyclic periods of hyperbaric oxygen and normoxic oxygen create a stress response by repeatedly increasing and decreasing the number of reactive oxygen species (ROS) in the tissues. ROS influence the signal transduction pathways of multiple growth factors, including those implicated in propagating angiogenesis. Thom (2011) published a concise review on this topic [6].

Both proposed mechanisms are also likely to play a role in delayed healing of lower extremity wounds and in our ischemia ulceration model in diabetic animals.

In the present study, no effect of HBOT on vasodilatory capability in ulcer tissue was observed. This is in contrast to our earlier work using a similar animal model in which we measured perfusion parameters on day 29 only [10]; in that study, because flow was restricted, and SO_2_ and rHB increased in HBOT animals, this suggested that HBOT induced vasoconstriction and improved tissue perfusion at the lower end of the capillary system. In the present study, on experimental day 28, these latter findings were not confirmed, i.e. in the present study, the overall effects of HBOT on tissue perfusion showed no significant differences over time when analyzed using a general linear repeated measures model.

SO_2_ values, measuring capillary venous oxygen saturation, showed a significant increase in the HBOT group only at a single time point, whereas rHB values showed a significant decrease in wounds of HBOT animals at several time points. Blood flow in the lower end of the capillary system was increased in 3 of the 6 time points but showed a significant decrease at the remaining two time points.

As HBOT has only a temporary effect on increasing tissue oxygenation, it could be argued that we missed these early effects on tissue oxygenation by performing measurements on the following morning, i.e. 8–20 h after the previous HBOT session had ended. Although this is a valid point, we deliberately avoided perfusion measurements immediately following HBOT as our pilot experiments showed substantial differences between animals, that seemed mainly attributable to the time that the measurements were made post-HBOT. Perfusion measurements take 5–10 min per animal. Therefore, the time post-HBOT varies considerably between the first and last animal when measurements start directly start after cessation of the HBOT session.

Another drawback of the perfusion measurements we performed is that we can only measure erythrocyte coupled oxygenation, and dispersion and speed of erythrocytes through the tissue. In view of the proposed mechanism of action of HBOT, i.e. dissolving oxygen under pressure in blood plasma brings oxygen (through plasma extravasation) from the small vasculature to bodily sites distant from vascularization, our perfusion measurements are not able to measure this. Real-time monitoring of oxygen levels in tissues of living animals on a 24-h basis is not possible, considering the large number of animals we included.

Therefore, an important aspect of HBOT, namely neovascularization to restore adequate blood flow, is not proven in this animal model. However, it has been demonstrated that HBOT induces angiogenesis and promotes neovascularization in wounds [28].

Excess inflammation and infection are consistent features of diabetes-impaired wound healing [29]. In this ischemia-reperfusion injury model, throughout reperfusion, leukocytes (after adhering to ischemic tissues) cause pathologic vasoconstriction and tissue damage by releasing proteases and free radicals [29]. In this study, the decreased dermal thickness (observed in the HBOT group on day 14) may reflect a more advanced wound healing in this group. Our inflammation data, slightly reduced on day 14 and significantly reduced on day 42, may also contribute to this view of advanced wound healing in HBOT animals, as it is tempting to speculate about an increased inflammation resolution in this group. The effects of HBOT on improvement of wound healing, together with resolution of inflammation, were also observed in our previous studies using this ulceration model, in which a heparin sulfate analogue was tested [24, 30]. Inflammation resolution one of the mechanisms by which HBOT may improve impaired wound healing. In addition, advancement of wound healing is also reflected in blood vessel condensation. Therefore, one might speculate a slight decrease in tissue perfusion in the HBOT group on day 14. However, crust formation precluded reliable SO_2_ and rHB measurements during the first two weeks of wound healing. On day 17, by which time the crust was detached from the wound in all animals, no significant differences were found in tissue saturation.

Collagen deposition and reorganization indicate the quality of wound healing [31]. Collagen deposition starts with procollagen synthesis. Subsequently, this procollagen is converted to collagen, and cross-linked to form a collagen matrix; this process is oxygen-dependent. In animal experiments, collagen deposition in a hyperoxic environment increased 3-fold compared with deposition in a hypoxic environment [32]. Improved ulcer breaking strength (biomechanical strength) is a powerful outcome of collagen biosynthesis, especially of cross-linked collagen biosynthesis. In the present study, the ulcer breaking strength showed a strong trend towards improvement following HBOT, as was also reported by Tuk et al. [10].

A limitation of the present study is that only female WAG/RijHsd rats were used; this implies that our findings may not be generalized to male rats or other rat strains.

In summary, not all evidence obtained from this animal model supports the hypothesis that HBOT has a positive effect on wound healing. Nevertheless, these data indicate that HBOT almost significantly improves tissue strength, and may have some effect on neovascularization at the later stages of wound healing.

## Supporting information

S1 Table Raw data file. (XLS)

S2 Table NC3Rs ARRIVE guidelines checklist. (PDF)
